# Correlation Between Mandibular Cortical Index and Primary Implant Stability

**DOI:** 10.7759/cureus.94777

**Published:** 2025-10-17

**Authors:** Atsushi Kobayashi, Keisuke Seki, Yoshimasa Takeuchi, Mika Furuchi, Ryosuke Murayama, Atsushi Kamimoto

**Affiliations:** 1 Department of Comprehensive Dentistry and Clinical Education, Nihon University School of Dentistry, Tokyo, JPN

**Keywords:** dental implant, digital panoramic radiography, implant stability quotient, mandibular cortical index, osteoporosis

## Abstract

Introduction

Adequate initial stability during implant placement is essential for successful implant therapy. Although elderly patients require special care for bone metabolic disorders, the prognosis of implant treatment in patients with osteoporosis remains unknown. This study aimed to statistically evaluate the mandibular cortical index (MCI) and implant stability quotient (ISQ).

Methods

This study included patients who visited the Nihon University School of Dentistry Dental Hospital, Japan, between 2012 and June 2025. The following information was extracted from the medical records: sex, age at implant placement, systemic diseases, implant size, and implant stability quotient (ISQ) data measured using a resonance frequency analyzer. The mandibular cortical index (MCI) classification of digital panoramic radiography (DPR) images taken after implant placement was evaluated in this study. The ISQ values and MCI data obtained were statistically analyzed at the implant level.

Results

The study included 79 patients (34 men and 45 women with a mean age of 60.3 ± 10.3 years at the time of implant placement) and 190 implants. The mean ISQ value was 71.6 ± 10.8 mm. The MCI classification at the implant level was as follows: Class I, 43 implants (72.8 ± 10.4); Class II, 100 implants (73.2 ± 10.4); and Class III, 47 implants (67.2 ± 11.2). Spearman's rank correlation coefficient revealed a weak negative correlation with ISQ values in MCI cases (-0.216, p < 0.01). However, no significant correlation was observed between the age at implant placement and ISQ values (-0.057, p > 0.05). A one-way analysis of variance with the ISQ value as the dependent variable revealed that the mean ISQ value for Class III was significantly lower (P < 0.01).

Conclusion

Class III in the MCI classification reflects bone demineralization. In this study, a significant decrease in the ISQ values was observed in the Class III group. Therefore, it was predicted that stability during implant placement would be reduced in cases involving porous mandibles. When assessing the prognosis of implant treatment, the morphology of the mandibular cortical bone should be carefully observed. For elderly patients and those at risk for osteoporosis, in particular, preoperative diagnosis of MCI is expected to be useful in predicting deterioration of bone quality at the implant site. This information can be used to adjust implant diameter and insertion torque and to determine the necessity of bone grafting procedures.

## Introduction

Dental implant treatment is currently recognized worldwide as the gold standard for replacing lost teeth [1,2] and has revolutionized oral rehabilitation [3]. Long-term follow-up studies have demonstrated high survival rates, making it a reliable treatment option for both patients and clinicians [4]. Accurate preoperative diagnosis and precise implant placement surgery are essential to ensure the success of the implant treatment [5]. Appropriate primary stability during implant placement is an important factor in achieving long-term osseointegration [6,7]. The stability of dental implants is generally evaluated using bone integration assessment methods that measure the implant stability quotient (ISQ) value via electronic resonance frequency analysis (RFA) [8,9]. Cortical bone density has been reported to influence changes in ISQ values [10].

Osteoporosis, a representative bone metabolic disease, affects 200 million people worldwide [11,12]. Osteoporosis is a chronic metabolic disease that often goes undetected because it is asymptomatic [13]. In recent years, the use of digital panoramic radiography (DPR) images for the morphological evaluation of the mandibular inferior cortical bone has been shown to be useful for osteoporosis screening [14,15]. Recent reviews have reported that osteoporosis, which causes a decline in bone quality and density, is a systemic disease that worsens the prognosis of implant treatment [16,17]. However, previous reports have indicated that dental implants can achieve high survival rates and stable clinical outcomes, even in postmenopausal women with osteoporosis [18]. This suggests that these issues remain unresolved. The question of how mandibular conditions affect implant stability and treatment prognosis remains unresolved in diagnostics. The success of implant treatment depends on closing this evidence gap.

This preliminary study aimed to statistically analyze the impact of a virtual bone quality assessment based on the mandibular cortical index (MCI) classification on implant stability, as measured by the ISQ value at the time of implant placement. Additionally, this study attempts to streamline the complex diagnostic methods necessary for successful implant treatment. Therefore, the null hypothesis for this study was that there was no correlation between the MCI class and ISQ scores. This retrospective descriptive epidemiological study was conducted at a single institution.

## Materials and methods

Study design

This retrospective cohort study investigated the implant stability index in patients who underwent implant placement surgery at the Nihon University School of Dentistry Dental Hospital, Tokyo, Japan. Patient information was collected from the hospital’s database. The Ethics Committee of the Nihon University School of Dentistry approved the research protocol (approval number: EP23D027). This study was conducted in strict accordance with the 2013 revision of the 1975 Declaration of Helsinki [19] and the STROBE (Strengthening the Reporting of Observational Studies in Epidemiology) guidelines [20]. The primary outcome of this study was to examine the correlation between MCI and ISQ values. The secondary outcome was to examine the correlation between the ISQ and implant data.

Study population

This study examined adult patients presenting at the Nihon University School of Dentistry Dental Hospital, aged 18 years or older at the time of implant treatment between January 2012 and June 2025, who were treated by a single implant specialist (KS). The inclusion criteria were as follows: availability of medical and dental records in the database, patients who underwent DPR imaging for implant treatment, and patients for whom the implant stability index was measured at the time of implant placement. The exclusion criteria included women who were pregnant or breastfeeding, cases in which a morphological diagnosis of the mandibular inferior could not be made due to artifacts or poor positioning in the acquired DPR images, patients with a history of mandibular resection or reconstruction surgery or bone-destructive lesions due to neoplastic lesions, and patients who had previously received radiation therapy to the head and neck region.

Demographic and implant data

The following survey variables were collected from the initial visit records: sex, date of birth, age at implant placement surgery, date of implant placement surgery, implant location, implant size, bone grafting, and early failure, which was defined as implant loss within six months postoperatively.

Evaluation of MCI

**Figure 1:**
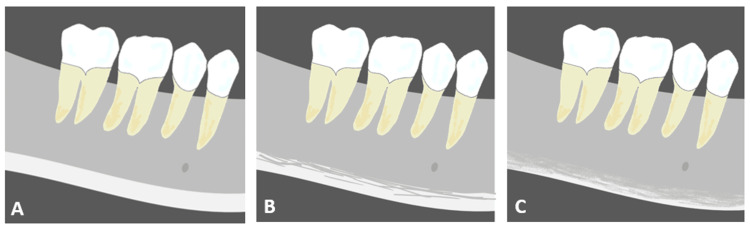
Schematic of MCI classification (A) Class I; (B) Class II; (C) Class III. Image Credits: Keisuke Seki, Author MCI: mandibular cortical index

All DPR images used in this study were obtained from preoperative implant surgery examinations and captured using the same X-ray imaging device (Veraviewepocs X700; J. Morita Corporation, Osaka, Japan). The output image data were evaluated using a diagnostic monitor (Eizo Corporation, Ishikawa, Japan). According to a report by Klemetti et al. [21], the cortical bone morphology of the mandibular inferior was classified into three types based on DPR images. Class I was characterized by a smooth inner surface of the cortical bone. Class II features an irregular inner surface of the cortical bone and linear resorption within it. Class III showed extensive linear resorption throughout the cortical bone (Figure 1). Two dentists (KS and AK) who had undergone prior classification training performed the MCI assessment twice, and the second assessment was used. Inter-rater reliability, as measured by the kappa coefficient, was examined for the two dentists who performed the assessments.

Evaluation of ISQ

All patients underwent screening diagnosis using DPR imaging. Diagnosis was made using dental X-ray CT imaging. Finally, a treatment plan was developed. A single implant specialist (K.S.) performed all the implant placement surgeries. After administering local anesthesia, the baseline thickness of the keratinized mucosa was clinically assessed using a periodontal probe. A conventional surgical procedure involving a crestal incision and gentle lifting of the full-thickness flap was performed. All implants were placed at the bone level or up to 0.5 mm subcrestal. The ISQ was measured immediately after implant placement using a resonance frequency analyzer. ISQ measurements were taken by securing a dedicated peg to the implant body and measuring the mesial and distal or buccal and lingual surfaces twice and recording the average value. ISQ values above 70 were taken to indicate good primary implant stability. Even when multiple implants were placed in the same patient during surgery, measurements were taken for each implant.

Statistical analysis

All statistical analyses were performed using EZR (Easy R) (Saitama Medical Center, Jichi Medical University, Tochigi, Japan) [22], a graphical user interface for R (version 4.0.0; The R Foundation for Statistical Computing, Vienna, Austria). Using the MCI classification and ISQ values obtained from the descriptive statistics, the correlations between each variable were tested. The normality of the data distribution for continuous variables (age, ISQ values, implant diameter, and implant length) was assessed using the Kolmogorov-Smirnov test, with P ≥ 0.05 considered indicative of a normal distribution. As none of these continuous variables were normally distributed (p < 0.05), Spearman's rank correlation coefficient was used to test for correlations. A one-way ANOVA was conducted with ISQ as the dependent variable for the three groups (C I, C II, and C III) of MCI. All tests were considered statistically significant at p < 0.05.

## Results

**Figure 2:**
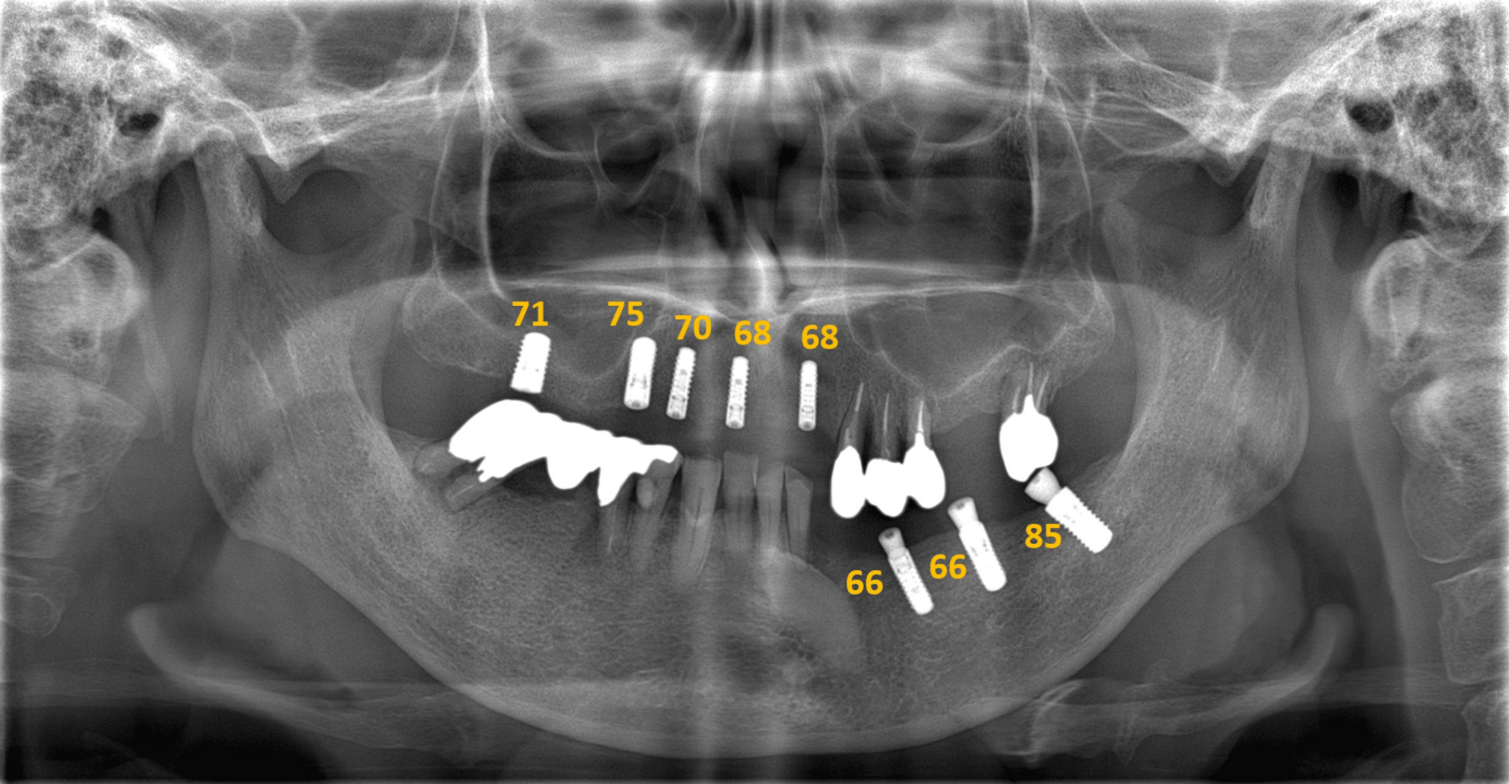
A 55-year-old woman with MCI Class III The numbers indicate ISQ values. The left mandibular canine and premolar region had the lowest value of 66 (without bone grafting). ISQ, implant stability quotient; MCI, mandibular cortical index

Of the 105 patients considered for the study, 79 met the inclusion criteria (34 men and 45 women) (Table 1). The average age of the patients at the time of implant placement was 60.3 ± 10.3 years. The study included 190 implants, with an average of 2.4 implants per patient. All implants had rough surfaces. The average length of the implants used was 10.0 ± 1.1 mm, and the average diameter was 4.0 ± 0.4 mm. There were 75 and 115 implant sites in the maxilla and mandible, respectively. Immediate placement after extraction was performed in seven cases, and bone augmentation was performed at the time of placement in 48 cases. Implant loss occurred in five cases within six months after placement. The mean ISQ value was 71.6 ± 10.8 (median = 73). The MCI classifications for each implant were 43 for Class I, 100 for Class II, and 47 for Class III (Table 2, Figure 2). The kappa coefficient representing inter-rater reliability was 0.64, indicating substantial agreement.

**Table 1:** Patient and implant data (79 patients) ISQ, implant stability quotient

Parameters	Mean ± S.D	Median (range)
Age (years)	60.3 ± 10.3	62.5 (27-79)
Implant diameter (mm)	4.0 ± 0.4	4.0 (3.3-5.0)
Implant length (mm)	10.0 ± 1.1	10.0 (7.0-13.0)
ISQ value	71.6 ± 10.8	73 (32-88)
Site (190 implants)	Frequency	Percentage
Upper anterior	28 implants	14.7%
Upper posterior	47 implants	24.7%
Lower anterior	6 implants	3.2%
Lower posterior	109 implants	57.3%
Immediate implant placement	7 implants	3.7%
Early implant failure	5 implants	2.6%
Bone augmentation (48 implants)	Frequency	Percentage
Developmental defects	25 implants	52.1%
Sinus floor elevation (crestal)	9 implants	18.8%
Split crest technique	14 implants	29.2%

**Table 2:** ISQ value by MCI class ^a^Mann-Whitney U test, ^b^Student's t-test, ^**^P < 0.01 ns, not significant; ISQ, implant stability quotient; MCI, mandibular cortical index

MCI	Implants	Bone augumentation	Immediate implant placement	Early implant failure	ISQ value		Correlation	p value	Significant difference
					Mean ± S.D.	Median (range)			
Class I	43	14	4	0	72.8 ± 10.4	75 (48-86)	Class I-II ^a^	0.904	ns
Class II	100	21	3	4	73.2 ± 10.4	75 (32-88)	Class II-III ^a^	0.000158	**
Class III	47	13	0	1	67.2 ± 11.0	69 (32-85)	Class I-III ^b^	0.00847	**

Table 3 presents the results of the one-way analysis of variance with the ISQ value as the dependent variable. The mean ISQ value for Class III was significantly lower (P < 0.01). This finding revealed that the means of at least one group differed from those of the other groups, and tests for differences between each of the three groups were examined. No significant difference was observed between Class I and Class II. However, significant differences were observed between Class II and Class III and between Class I and Class III.

**Table 3:** Correlation between implant stability quotient (ISQ) and patient data ^**^P < 0.01, ^†^Spearman's rank correlation coefficient, ^‡^Kendall's rank correlation coefficient ns, not significant; MCI, mandibular cortical index

Patient data	P value	Significant difference	*r* or* τ*
^†^Age	0.434	ns	-0.057
^†^Implant diameter	0.00000701	**	0.319
^†^Implant length	0.1606	ns	0.102
^‡^MCI: Class III (Class III: 1, Class I and II: 0)	0.0001567	**	-0.229
^‡^Bone augmentation (yes: 1, no: 0)	0.0002877	**	-0.220
^‡^Early implant failure (yes: 1, no: 0)	0.3619	ns	0.055
^‡^Immediate implant placement (yes: 1, no: 0)	0.008917	**	-0.158

Spearman's rank correlation coefficient was used to analyze implant stability and patient data. A weak negative correlation was observed between ISQ values and MCI (-0.216, P < 0.01), but no significant correlation was found between age at implantation and ISQ values (-0.057, P > 0.05). The diameters and lengths of the implants were found to be non-normally distributed. Spearman's rank correlation coefficients were then calculated for each. There was a weak correlation between implant diameter (0.319, P < 0.01) and ISQ, but no significant difference was observed in implant length (0.102, P = 0.161). Kendall's correlation coefficient revealed weak correlations between ISQ values, MCI C3, bone augmentation, and immediate implant placement. No significant difference was observed in early implant loss.

## Discussion

This study retrospectively examined the association between the MCI and implant stability. As a result, it was revealed that the ISQ value was significantly lower in the Class III group of the MCI classification. Additionally, Spearman's rank correlation showed a weak negative correlation between MCI and ISQ values. This result rejects the null hypothesis that there is no correlation between the MCI class and ISQ values. Class III of the MCI classification, which indicates mandibular cortical bone rarefaction, has been suggested to be associated with reduced primary stability during implant placement.

Primary stability during implant placement is critical for achieving long-term osseointegration and significantly influences the clinical prognosis [23]. Previous reports have demonstrated that ISQ values are influenced by cortical bone density [10,24], and the results of this study support this finding. Class III MCI is characterized by cortical bone thinning and rupture patterns in the mandible associated with osteoporosis. This type may serve as an indirect indicator of bone metabolic abnormalities [14,15,25]. Therefore, this study considered MCI Class III as a surrogate marker for osteoporosis. It is generally known that the mandible, which has a denser bone structure, has a shorter implant healing period than the maxilla [26]. This study demonstrated that the ISQ value was significantly lower in the MCI Class III group. This suggests that ISQ may be a useful prognostic factor when planning implant treatment for patients with osteoporosis or deteriorating bone quality. 

The MCI assessment showed substantial agreement, with a Kappa coefficient of 0.64. However, inter- and intra-observer variability may still have influenced the results. In the future, improving the reproducibility of diagnoses will require the use of more standardized evaluation criteria and automated artificial intelligence (AI)-based assessments. However, no significant correlation was observed between age and ISQ. This suggests that aging may not be the primary cause of reduced implant stability. Rather, individual variations in bone quality and the presence of metabolic diseases are important factors [7]. Conversely, since implant diameter showed a weak positive correlation with ISQ, factors influencing stability should include not only bone quality but also implant design and size selection [7,27]. From a clinical perspective, preoperative MCI assessment using DPR imaging is straightforward and minimally invasive. It is also a useful adjunctive diagnostic tool for evaluating bone quality [28]. For elderly patients and those at risk for osteoporosis, in particular, preoperative diagnosis of MCI is expected to be useful in predicting the deterioration of bone quality at the implant site. This information can be used to adjust the implant diameter and insertion torque and to determine the necessity of bone grafting procedures. Furthermore, careful planning of treatment strategies is required for cases diagnosed with MCI Class III because of the difficulty in achieving primary stabilization.

This study had several limitations. First, this was a retrospective, single-center study; therefore, bias due to case selection and recording limitations cannot be ruled out. Second, the outcomes of this study were limited to the ISQ immediately after implant placement. However, the relationship between these factors and long-term osseointegration maintenance or implant survival rates remains unclear. Even in Class III cases with poor primary stability, there is potential to improve the long-term prognosis through appropriate bone augmentation and treatment planning. Future validation through longitudinal studies is required. The third limitation was the lack of detailed patient information regarding osteoporosis diagnoses and medication histories, such as bisphosphonate use. This prevented a direct assessment of the relationship between MCI and systemic bone metabolism. Finally, this preliminary study examined the ISQ, which included both the maxilla and the mandible. We designed the study to estimate the bone quality predicted by MCI as representative of each individual. However, to identify general principles, we should have examined correlations using only mandibular samples.

## Conclusions

This study is significant because it is the first to demonstrate the relationship between MCI and implant stability in a Japanese population. The significant association between MCI Class III and reduced ISQ justifies the use of DPR imaging in preoperative diagnosis and demonstrates its potential for clinical implementation. However, future multicenter collaborative and prospective cohort studies are essential to verify the diagnostic accuracy and its association with long-term prognosis. In the future, the accuracy of predicting risks associated with implant treatment is expected to improve with the construction of an evaluation model integrating MCI imaging diagnostic indicators and bone density measurements obtained via CT.
